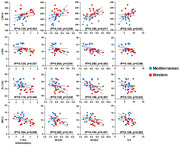# Relationships between Circulating Monocyte and Brain Transcriptional Profiles in Nonhuman Primates Consuming Mediterranean versus Western Like Diets

**DOI:** 10.1002/alz.093067

**Published:** 2025-01-03

**Authors:** Thomas C. Register, Brett M. Frye, Jacob D. Negrey, Corbin S.C. Johnson, Noah Snyder‐Mackler, Suzanne Craft, Thomas J. Montine, Carol A. Shively

**Affiliations:** ^1^ Wake Forest University School of Medicine, Winston‐Salem, NC USA; ^2^ Wake Forest Alzheimer’s Disease Research Center, Winston‐Salem, NC USA; ^3^ Emory and Henry College, Emory, VA USA; ^4^ University of Washington, Seattle, WA USA; ^5^ Arizona State University, Tempe, AZ USA; ^6^ University of Washington School of Medicine, Seattle, WA USA; ^7^ Stanford University, Stanford, CA USA

## Abstract

**Background:**

Mediterranean diets may reduce Alzheimer’s disease (AD) risk and preserve cognitive function relative to Western diets by protecting against inflammation. In a long term controlled randomized trial of Mediterranean vs. Western diet consumption in cynomolgus macaques (*Macaca fascicularis*), difficult to conduct in humans, we found significant anti‐inflammatory effects of Mediterranean diet on circulating monocyte and brain temporal cortex transcriptional profiles. Here we examine relationships between monocyte and temporal cortex gene expression profiles.

**Methods:**

38 middle‐aged (mean = 12 years of age) female macaques were fed Western or Mediterranean diets for 2.5 years in a randomized nonhuman primate trial. RNAseq was used to assess transcriptional profiles in CD14+ monocytes after 15 months of diet consumption while temporal cortex was obtained after 2.5 years.

**Results:**

Cortex expression of cyclin dependent kinase 14 (*CDK14*), a proinflammatory regulator, was lower in the Mediterranean group. Six other cortex transcripts [i.e., “lunatic fringe” (*LFNG*), mannose receptor C type 2 (*MRC2*), solute carrier family 3 member 2 (*SLCA32*), butyrophilin subfamily 2 member A1 (*BTN2A1*), katanin regulatory subunit B1 (*KATNB1*), and transmembrane protein 268 (*TMEM268*)] higher in the Mediterranean group were associated with anti‐inflammatory/neuroprotective pathways. Monocyte patterns of gene expression predicted expression of these targets in the brain, with consistent anti‐inflammatory effects of the Mediterranean Diet relative to the Western Diet (Figure 1). Monocyte transcripts associated with inflammation, including proinflammatory components of the constitutive transcriptional response to adversity (CTRA, Cole et al. 2015), *NFKB1*, *NFKB2*, and *IL6*, were positively associated with temporal cortex transcript level of the pro‐inflammatory gene *CDK14* (all p’s≤0.0?). In general, these proinflammatory monocyte transcript levels were negatively correlated with temporal cortex anti‐inflammatory transcripts *LFNG*, *SLC3A2*, and *MRC2* (8 of 12 correlations p<0.05; 11 of 12 correlations p<0.10), consistent with an overall anti‐inflammatory effect of the Mediterranean diet.

**Conclusion:**

Circulating monocyte gene expression profiles correlated with temporal cortex transcript levels consistent with anti‐inflammatory effects of the Mediterranean Diet relative to the Western Diet, suggesting that peripheral inflammation may promote neuroinflammation. Interventions including diet to target inflammation may have a role in prevention or treatment of systemic as well as neuro‐inflammation and neuropathology.